# The Barbarous Lunacy Law of Texas

**Published:** 1910-11

**Authors:** 


### EDITORIAL DEPARTMENT.

### THE BARBAROUS LUNACY LAW OF TEXAS.

In Texas, the "crime" of lunacy is the only unbailable "offense." A man may commit murder, homicide, arson, rape, theft, burglary, or what not, and except in rare cases he can get release on bond, or by habeas corpus, pending trial. But let one be denounced to the court as being even suspected of lunacy—and of being dangerous—(or inoffensive)—and any one can so denounce another—the victim is arrested by the police by order of the court, hauled up before the court—like a thief—tried by a jury picked up on the street—like a thief, is prosecuted by the county or district attorney—like a thief, must be defended by a lawyer—like a thief, is acquitted—or convicted—like a thief—and, if convicted, he is sentenced—to the asylum (an "asylum" is—well, see your dictionary)—and if a criminal (and in law "an insane person can not commit a crime; if he is insane, it is not crime, only sane persons can commit crime."—Hurt) he is locked up, solitary confinement. Henry Purnell, an inmate of the Texas Insane Asylum at Austin, killed the Superintendent. He was locked up seventeen years, and died of consumption in his solitary cell.

Of course, I do not contend that an insane person, even the harmless, should be bailed; what I mean is that pending the trial for lunacy (?) he should not be locked up.

The dreadful fate of a suspect is illustrated by the case of Col. John Dowell, a prominent lawyer, President of the Austin Bar Association, in fact. He was under sentence to the penitentiary for assault to murder, and was himself badly shot up in the encounter. Pending appeal, he was out on bond. But some one denounced him as a dangerous lunatic, who should be locked up—and he was arrested and tried in the manner above described, and convicted of lunacy, "but not to be locked up," was the verdict of the jury, and he was discharged. But pending the trial (which extended over several weeks, at intervals) he was in jail—like a thief, denied bail, taken from his home, family and business, put to the expense of defense, subjected to the greatest humiliation and mental distress—and—he has no recourse. He is now practicing law in Houston—one of the ablest lawyers in the State. And he had to pay the court costs, I suppose, inasmuch as others who are so denounced, arrested, tried and convicted—even the poorest and most harmless do. I have before me the bill of costs in the case of an old, harmless, demented woman, a pauper, I understand. The bill of costs in such cases is a good thing for the court officers. I should state that on the warrant issued for the arrest of this old woman (and on all such warrants) is printed "offense," and filled in in ink,—"lunacy."

BILL OF COSTS IN LUNACY TRIAL.

*Clerk's and Sheriff's Fees.*—Docketing cause, 25c.; filing papers, 60c.; entering appearances, 15c; swearing witnesses, 20c.; oaths and certificates, 50c; subpoenas, 25c.; additional names, 15c; empaneling jury, 50c; entering judgment, two additional, 75c.; entering indictment testimony, $1; commitment, $1; taxing cost, 25c; issuing attachments transcript, $2.50; serving capias and mileage, $1; summoning witnesses and mileage, $1; jury fee, 50c; commitment, $1.50; trial fee, $3; jury fee, $3; attorney fee, $5; Dr., $10. Total, $33.10.

And this old woman had to pay it.

Are we civilized? To a jury of tinkers and tailors and candlestick makers—often illiterate, is left the determination of questions that often baffle the ablest alienists. This law should be repealed or amended by the next Legislature. What we need is a Lunacy Commission, of the ablest and most experienced men in the State. Questions of law are decided by legal experts, the highest courts. Questions of lunacy are decided by illiterate laymen. It is inhuman, barbarous, and a disgrace to our boasted civilization. There should be a medical court for such cases.

